# Increases in Alcohol and Cannabis Use Associated with Deteriorating Mental Health among LGBTQ2+ Adults in the Context of COVID-19: A Repeated Cross-Sectional Study in Canada, 2020–2021

**DOI:** 10.3390/ijerph182212155

**Published:** 2021-11-19

**Authors:** Trevor Goodyear, Allie Slemon, Chris Richardson, Anne Gadermann, Travis Salway, Shivinder Dhari, Rod Knight, Emily Jenkins

**Affiliations:** 1 School of Nursing, University of British Columbia, Vancouver, BC V6T 2B5, Canada; trevor.goodyear@ubc.ca (T.G.); allie.slemon@ubc.ca (A.S.); dharis@camosun.bc.ca (S.D.); 2 British Columbia Centre on Substance Use, Vancouver, BC V6Z 2A9, Canada; rod.knight@bccsu.ubc.ca; 3 School of Population and Public Health, University of British Columbia, Vancouver, BC V6T 1Z3, Canada; chris.richardson@ubc.ca (C.R.); anne.gadermann@ubc.ca (A.G.); 4 Centre for Health Evaluation and Outcome Sciences, St. Paul’s Hospital, Vancouver, BC V6Z 1Y6, Canada; 5 The Human Early Learning Partnership, University of British Columbia, Vancouver, BC V6T 1Z3, Canada; 6 British Columbia Centre for Disease Control, Vancouver, BC V5Z 4R4, Canada; travis_salway@sfu.ca; 7 Centre for Gender and Sexual Health Equity, Vancouver, BC V6Z 1Y6, Canada; 8 Faculty of Health Sciences, Simon Fraser University, Burnaby, BC V5A 1S6, Canada; 9 Department of Medicine, University of British Columbia, Vancouver, BC V6T 1Z3, Canada

**Keywords:** LGBT people, mental health, suicide, substance use, alcohol, cannabis, COVID-19, public health, survey

## Abstract

Lesbian, gay, bisexual, trans, other queer, and Two-Spirit (LGBTQ2+) people are particularly at risk for the psycho-social consequences of the COVID-19 pandemic, though population-tailored research within this context remains limited. This study examines the extent of, and associations between, increased alcohol and cannabis use and deteriorating mental health among LGBTQ2+ adults in Canada during the COVID-19 pandemic. Data are drawn from LGBTQ2+ respondents to a repeated, cross-sectional survey administered to adults living in Canada (May 2020–January 2021). Bivariate cross-tabulations and multivariable logistic regression models were utilized to examine associations between increased alcohol and cannabis use, and self-reported mental health, overall coping, and suicidal thoughts. Five-hundred and two LGBTQ2+ participants were included in this analysis. Of these, 24.5% reported increased alcohol use and 18.5% reported increased cannabis use due to the pandemic. In the adjusted analyses, increased alcohol use was associated with poor overall coping (OR = 2.28; 95% CI = 1.28–4.07) and worse self-reported mental health (OR = 1.98; 95% CI = 1.21–3.25), whereas increased cannabis use was associated with suicidal thoughts (OR = 2.30; 95% CI = 1.16–4.55). These findings underscore the need for population-tailored, integrated substance use and mental health supports to address interrelated increases in alcohol/cannabis use and worsening mental health among LGBTQ2+ adults, in the context of the COVID-19 pandemic and beyond.

## 1. Introduction

The COVID-19 pandemic and associated public health measures are poised to deleteriously impact population-level mental health, as seen in research showing increases in overall levels of anxiety, depression, and suicidal thinking [1,2,3]. The pandemic and its social and economic consequences also have noteworthy implications related to substance use. Specifically, people are reporting using substances to cope with new and/or intensified experiences of stress, fear, boredom, and isolation; and/or to facilitate experiences of pleasure and connectedness during these challenging times [4,5]. Although individuals may use a variety of substances to manage the psycho-social impacts of the pandemic, those that are legal and regulated are used more widely [4,5,6]. Rates of alcohol and cannabis use (especially where legalized) have indeed risen alongside the COVID-19 pandemic [7,8,9,10,11,12]. Use of either (or both) of these substances, if frequent and/or intensive, has potential to heighten risks for alcohol and cannabis-related harms, including dependence and adverse mental health outcomes [13,14]. Within the pandemic context, there are also notable concerns about harmful patterns of alcohol/cannabis use being associated with concerning global trends, such as using substances to cope with disrupted socialization, economic insecurity, and loss and grief related to the pandemic, as well as possibly fueling increased rates of violence [7,10,12,15,16].

The mental health and substance use impacts of the pandemic are likely to be unevenly distributed along a social gradient, with structurally vulnerable populations experiencing the greatest risks and worst health outcomes [17]. Drawing on prior theorizing from Bourgois and colleagues [18], we define “structural vulnerability” as the inequitable burden of risk for negative health outcomes stemming from individuals’ locations within intersecting power hierarchies, which create and maintain barriers to determinants of good health. Lesbian, gay, bisexual, trans, other queer, and Two-Spirit (LGBTQ2+) people represent one such structurally vulnerable population. (Here, we use the term “trans” to encompass transgender, non-binary, genderqueer, agender, and other gender non-conforming identities and experiences; and “Two-Spirit” as an umbrella term and community organizing tool encapsulating Indigenous conceptualizations of diverse genders and sexualities [19]). LGBTQ2+ people constitute a group for whom there have been calls for proactive and targeted public health responses to mitigate the mental health and substance use consequences of the COVID-19 pandemic [20,21,22]. Even prior to the pandemic, LGBTQ2+ people were reporting higher rates of substance use (including alcohol and cannabis) compared to cisgender and heterosexual people [23,24,25,26]. LGBTQ2+ people are also known to face disproportionately high rates of negative mental health outcomes, including diagnosed depression and anxiety [27,28,29], as well as experiences of self-harm and suicidality [30,31]. Mental health and substance use inequities such as these may be exacerbated by pandemic-driven infringements upon social determinants of good health, which are poised to disproportionately impact LGBTQ2+ communities. For example, LGBTQ2+ people have been found to be at greater risk of harm secondary to the socio-economic consequences of COVID-19, including cuts to work hours and under/unemployment [32,33]. Also, LGBTQ2+ individuals must now contend with public health-mandated disruptions in access to safe and supportive LGBTQ2+ community spaces (e.g., gathering places, leisure groups) that are known to have protective effects for mental health and overall wellbeing, particularly for individuals who are not meaningfully supported–or, worse, who are marginalized–by cisgender and heterosexual peers [34,35,36]. Already, several studies have underscored that disconnection from community, alongside other COVID-19 stressors, are contributing to adverse mental health impacts among LGBTQ2+ people, including lower levels of hope for the future, and increases in alcohol and cannabis consumption [21,37,38,39].

Previous research has pointed to “negative reinforcement” relationships between intensive substance use and mental ill health [40,41]. For some people, experiences of psycho-social stress can contribute to more frequent and heavier consumption of alcohol and/or cannabis, which can then intensify feelings of mental distress [13,14]. Yet, the nature of these relationships within LGBTQ2+ communities amid the pandemic remains underexplored, as recent epidemiological studies have had limitations with respect to capturing data and/or conducting sub-analyses related to LGBTQ2+ identities and experiences [7,8,10]. And, although contemporary studies investigating experiences of and disparities in worsening mental health and psycho-social stress among LGBTQ2+ people are emerging [21,38,42], the relationships between mental health and patterns of substance use, including alcohol/cannabis use, that may arise due to the pandemic remain un(der)explored within this population context. Targeted investigation is needed to address these knowledge gaps, and to inform policy and practice responses to the existing–and potentially evolving–burden of drug and mental health-related harms faced by LGBTQ2+ people. Accordingly, the aims of this paper are to describe the extent of increased alcohol/cannabis use among LGBTQ2+ adults in Canada during the COVID-19 pandemic, and then to examine associations between increased alcohol/cannabis use and selected adverse mental health outcomes–as measured through self-reported mental health, overall coping, and suicidal thoughts.

## 2. Materials and Methods

### 2.1. Study Overview

This study draws on data from a repeated cross-sectional monitoring survey, “Assessing the Impacts of COVID-19 on Mental Health”, which examines the self-assessed mental health impacts of COVID-19 among adults 18 years of age and older living in Canada. This survey was designed by our research team at the University of British Columbia, and is administered in partnership with the Canadian Mental Health Association (CMHA) and the Mental Health Foundation in the United Kingdom. Approval for this study was granted by the University of British Columbia Behavioural Research Ethics Board (H20-01273). Participants provided online consent in advance of completing the survey and were remunerated with a small honorarium.

The present study is informed by established theoretical frameworks, including the minority stress model [43] and psychological mediation framework [44]. These frameworks provide evidence-based rationale for understanding how stressors may impact LGBTQ2+ people differently [21]. Indeed, these theories offer insights into the compounding effects of overall increased stress exposure (e.g., related to anti-LGBTQ2+ stigma), as well as the ways in which this inequitable burden of stress aggravates emotional dysregulation and shapes the associated cascade of responses, including using substances to cope, that directly or indirectly influence mental health challenges [43,44].

### 2.2. Data Collection

This study draws on three rounds of data that were collected at four-month intervals between May 2020 and January 2021, alongside a rapidly evolving national landscape related to COVID-19 and associated public health responses [45]. Round 1 data collection (14–19 May 2020) occurred during a “re-opening” phase of the pandemic, at which time public health restrictions began to ease alongside stabilization of the initial surge of COVID-19 cases in Canada. Round 2 data were collected (14–21 September 2020) as case numbers started to rise again following the summer period, during which outdoor socializing contributed to reductions in virus transmission. Round 3 data were collected (22–28 January 2021) following the winter holiday season, which coincided with a rapidly increasing COVID-19 case count and corresponding increasing public health restrictions across Canada.

The online surveys were distributed by Maru/Matchbox, a national polling vendor that maintains a panel of approximately 125,000 members across Canada [46]. Maru/Matchbox randomly sampled panel members from across all Canadian provinces and territories. Selection was stratified based on Canadian Census-informed socio-demographic characteristics (age, gender, household income, region), with adjustments for response propensity, to yield a nationally representative sample according to these characteristics. Response-to-invitation rates were 32% at Round 1, 36% at Round 2, and 36% at Round 3. For participants who were sampled in multiple survey rounds, we retained data for the first round of participation and excluded subsequent survey data, thereby ensuring that the survey rounds contained independent and non-overlapping samples.

### 2.3. Survey Development and Measures

The development of survey items was informed by a monitoring survey commissioned by the Mental Health Foundation in 2020. Item development for this parent survey was grounded in research on the mental health impacts of past pandemics, and was refined through a citizens’ jury participatory process that solicited input from people with lived experience of mental health conditions. For the present survey, items were modified and added to reflect the Canadian context. Surveys were available in Canada’s two official languages: English and French.

This survey included detailed socio-demographic questions, incorporating items about gender, sexual orientation, race/ethnicity, socio-economic status, and mental health and disability status. In Round 1, gender was assessed through the question, “Which gender do you most identify with?” with response options: “Man”; “Woman”; “Transgender woman/trans woman”; “Transgender man/trans man”; “Non-binary”; “Two-Spirit”; “Not listed”; and “Prefer not to answer”. Based on feedback from the study team, this item was modified for Rounds 2 and 3, where gender was assessed by asking, “Which gender do you most identify with?” with response options: “Female”; “Male”; “Non-binary”; “Two-Spirit”; “Not listed”; and “Prefer not to answer”. Here, participants were also asked to indicate their assigned sex at birth, with binary response options “Female” and “Male”. Comparisons between measures of current gender and sex assigned at birth were then used to identify binary trans participants (i.e., if a participant identified their current gender as “Female” and reported that they were assigned “Male” at birth, we classified them as trans). Sexual orientation was broadly assessed through the question, “Do you identify as being LGBT2Q+ (lesbian, gay, bisexual, trans, Two-Spirit, queer, etc.)?” Participants who answered either “Yes” or “Unsure” were classified as LGBT2Q+, with a view to include individuals who may be “questioning” their sexual orientations and/or holding fluid sexual identities. Race/ethnicity was measured by asking participants to identify their “ethnic origin”. Participants who identified only European origins were classified as non-racialized, whereas those who identified one or more non-European origins were classified as racialized persons. All participants who identified Indigenous origins were classified as Indigenous, regardless of other reported origins.

The survey included additional items related to alcohol/cannabis use and mental health, which we used, respectively, as our primary exposure and outcome variables. Substance use was assessed by asking participants to “indicate how your use of any of the following has been impacted by the COVID-19 pandemic”, including “Drinking alcohol” and “Use of cannabis products”. Response options included “More”, “Less”, “No change”, “Not applicable”, and “Prefer not to say”. Respondents who indicated “More” were classified as having increased their use of the respective substance, whereas those who indicated “Less”, “No change”, and “Not applicable” were classified as not having increased their substance use. In terms of mental health, we assessed coping through the question, “Overall, how well do you think you are coping with stress related to the COVID-19 pandemic?” Response options “Not very well” and “Not well at all” were classified as poor coping, whereas responses “Very well” and “Fairly well” were classified as not poor coping. Self-reported change in mental health was also assessed by asking participants, “Compared to before the COVID-19 pandemic and related restrictions in Canada, how would you say your mental health is now?” Response options “Slightly worse now” and “Significantly worse now” were classified as experiencing worse mental health, whereas responses “Significantly better now”, “Slightly better now”, and “About the same” were classified as not experiencing a deterioration in mental health. Suicidal thoughts were then assessed through a question asking whether participants had “Experienced suicidal thoughts/feelings” within the past two weeks.

### 2.4. Data Analysis

The current analysis is restricted to the sub-sample of survey respondents classified as LGBTQ2+. Descriptive statistics were used to examine the socio-demographics and prevalence of increased alcohol and cannabis use for each of the three rounds of data collection. We then pooled these data to increase our sample size when examining the relationship between increased alcohol/cannabis use and mental health. With the pooled data, bivariate cross-tabulations with chi-squared tests were used to identify bivariate associations between increased use of alcohol/cannabis and each of the mental health outcomes (coping, change in mental health, and suicidal thoughts). Next, separate multivariable logistic regression models were used to quantify the independent association(s) between alcohol/cannabis use and each of the mental health outcomes, with adjustment for *a priori*-specified socio-demographics. These socio-demographic variables–age, household income, race/ethnicity, gender identity, and having a pre-existing mental health condition–were selected based on previous literature suggesting their links to substance use and mental health [9,17,21,22]. Survey round was included as an additional covariate to control for the self-assessed impact of pandemic phase on mental health. *p*-values < 0.05 were considered statistically significant. Data were analyzed using SPSS Version 27 [47].

## 3. Results

Of the 9061 completed surveys, 1993 (22%) were from respondents who participated in more than one survey round. This resulted in a final sample of 7068 unique participants, of which 502 (7.1%) were classified as LGBTQ2+. Comparisons between the mental health and substances use practices of LGBTQ2+ respondents and those of non-LGBTQ2+ respondents are explored in depth in a forthcoming analysis by our team [48], whereas the current analysis addresses population-specific knowledge gaps by narrowing in on LGBTQ2+ experiences. Socio-demographic characteristics for the LGBTQ2+ respondents included in this investigation are provided in Table 1. We present these data at both the aggregate level and stratified by survey round to provide a fuller picture about the self-assessed impacts of the pandemic at various times. Among all survey participants, 24.5% reported increased alcohol use and 18.5% reported increased cannabis use due to the pandemic. Moreover, 22.1% of participants reported experiencing poor overall coping with stress related to the pandemic, 47.4% indicated that their mental health had worsened when compared to before COVID-19, and 16.9% endorsed having had suicidal thoughts within the two weeks prior to being surveyed.

As indicated in Table 2, the associations between increased alcohol use and overall coping, change in mental health, and suicidal thoughts were all statistically significant (chi-squared = 10.8, *p* < 0.01, chi-squared = 18.5, *p* < 0.01, chi-squared = 10.4, *p* < 0.01, respectively), as were the associations between increased cannabis use and overall coping, change in mental health, and suicidal thoughts (chi-squared = 4.2, *p* < 0.05, chi-squared = 11.8, *p* < 0.01, chi-squared = 39.3, *p* < 0.01, respectively). The proportion of participants who reported increasing their alcohol/cannabis use was higher among those who reported adverse mental health outcomes, relative to those who did not (see Table 2).

The results of the three separate multivariable logistic regression models examining the independent association(s) between increased alcohol/cannabis use and each of the mental health outcomes with adjustment for the *a priori*-specified socio-demographics and survey round are presented in Table 3. Increased alcohol use was associated with an increased likelihood of reporting poor overall coping (odds ratio (OR) = 2.28; 95% confidence interval (CI) = 1.28–4.07) and an increased likelihood of reporting a deterioration in self-reported mental health (OR = 1.98; 95% CI = 1.21–3.25), whereas increased cannabis use was associated with a greater likelihood of reporting suicidal thoughts (OR = 2.30; 95% CI = 1.16–4.55).

## 4. Discussion

This paper draws on three rounds of data from a nationally representative, cross-sectional monitoring survey—assessing the mental health impacts of the COVID-19 pandemic in Canada—to examine associations between increased alcohol/cannabis use and select adverse mental health outcomes among the sub-sample of LGBTQ2+ adult survey respondents. Findings illustrate that approximately one in five LGBTQ2+ adults surveyed increased their consumption of alcohol and/or cannabis during the pandemic. Our unadjusted analyses indicated that increased alcohol/cannabis use was significantly associated with each of our survey’s measures of poor mental health. Even after adjustment for social determinants of mental health and substance use, our multivariable logistic regression models yielded significant associations between increased alcohol use and poor overall coping and worse self-reported mental health, and between increased cannabis use and suicidal thoughts. These findings corroborate and extend upon emerging data documenting the disproportionate mental health and substance use impacts of the pandemic on LGBTQ2+ adults [21,37,38,42], signaling a need for focused public health responses to mitigate these ongoing challenges.

It is neither uncommon nor unexpected to see population-level increases in substance use during times of crisis and stress [15], as is increasingly being demonstrated in the current COVID-19 context. Indeed, our findings that approximately 25% of LGBTQ2+ adults reported having recently increased their use of alcohol are similar to estimates from other international surveys, such as 27% increases in alcohol consumption among Australian adults [49], and 17% increases among adults in the United Kingdom [8]. Similarly, our findings of about 19% sample-wide (i.e., including people who do and do not consume cannabis in the denominator) increases in cannabis use align with the available literature showing heightened use among regular and/or medicinal cannabis users in the United States, Netherlands, and Belgium, in the context of the pandemic [6,12,50]. Of note, the substantial proportion of LGBTQ2+ adults reporting increased alcohol/cannabis use in this study also did not decrease appreciably nor consistently across rounds of our survey. Rather, reported trends in alcohol/cannabis use seemed to coincide with an inverse “V” pattern in which increased alcohol/cannabis was most prevalent during the second survey round, suggesting a possible rising trend in substance use in the earlier months of the pandemic (i.e., between spring and fall 2020), followed by a decreasing trend between fall 2020 and winter 2021. Here, our findings also revealed significant associations between intensified patterns of alcohol/cannabis use and deteriorating mental health–findings that are expressly disconcerting because LGBTQ2+ people have been identified as a population susceptible to worsening of mental health and substance use outcomes during the pandemic, due to ongoing societal stigma and isolation [51,52]. Thus, although it has elsewhere been documented that LGBTQ2+ people may increase consumption of alcohol/cannabis to manage stress in the context of COVID-19 [37], our findings caution that this increased use may coincide with unintentional deteriorations in mental health. This may be particularly so for alcohol, given past theorizing on the combination of strong positive outcome expectancies (e.g., increased sociability, disinhibition) and low negative expectancies (i.e., that one will experience harms) related to alcohol consumption within this group, due to potentially more permissive social norms about alcohol/substance use within LGBTQ2+ communities [44]. Additional longitudinal and qualitative investigation is needed to further characterize evolving and potentially harmful patterns of use such as these, and to inform mental health and substance use care responses.

Findings from this study underscore the need for targeted supports for LGBTQ2+ people experiencing worsening mental health alongside increased alcohol/cannabis use. The significant association between increased cannabis use and suicidal thinking within this study population is noteworthy, especially since previous studies have tended to only associate intensive and/or chronic cannabis use with suicidality [53,54]. Our findings associating increased alcohol use with elevated odds of experiencing poor overall coping and worse self-reported mental health are also concerning, and align with emerging research linking increased alcohol consumption with depressive mental health impacts during the COVID-19 era [8,49]. The distinct and prolonged stressors occurring during the pandemic are critical to consider here. Indeed, using substances as a coping strategy is associated with increased risk for experiencing acute and longer-term harms [55], such as experiencing drug dependence and/or substance use disorders, with people who are LGBTQ2+ already overrepresented in these data [23,24,25,26]. Given the interconnected nature of our findings related to increased alcohol/cannabis use and deteriorating mental health, we join others in arguing that integrated approaches to mental health and substance use policy, practice, and care may hold particular promise for supporting LGBTQ2+ people in this health context [56]. Such responses must be delivered in real-time and throughout future phases of the pandemic, and are likely to have optimal impact if co-designed with LGBTQ2+ communities and implemented in ways that are safe, welcoming, and accessible for members of this population [52].

In this study, associations between increased alcohol/cannabis use and certain measures of worsened mental health persisted after adjusting for pre-existing mental health conditions. This is chiefly relevant here, given that pre-existing mental health conditions and substance use were highly prevalent within our sample and among LGBTQ2+ people more generally [27,28,29], and that having a pre-existing mental health conditions is associated with heightened susceptibility to increased substance use and adverse mental health impacts in the context of the COVID-19 pandemic [9,52,57,58]. Our analyses also uncovered differential mental health impacts by household income, gender, age, and race/ethnicity, thereby substantiating concerns that LGBTQ2+ adults experiencing intersecting structural vulnerabilities may be particularly hard hit by the pandemic’s mental health ramifications [33,52,59]. Alarmingly, Indigenous respondents to this survey had nearly four-fold higher odds of having suicidal thoughts relative to non-racialized respondents. These findings underscore demand for health research and practice responses that specifically attend to the health and healthcare access needs of Two-Spirit and other LGBTQ+ Indigenous Peoples, including, for instance, population-tailored and culturally safe services that fully account for this group’s cultural, gender, and sexual identities [60,61]. Also of note, our study found that younger LGBTQ2+ adults (ages 18–34 and 35–54) had greater odds of experiencing poor overall coping and suicidal thoughts when compared to older respondents. These findings, particularly with LGBTQ2+ adults ages 18–34, align with elsewhere-documented hypotheses that younger LGBTQ2+ people may be disproportionately impacted by the psycho-social consequences of the pandemic, including due to being more likely to live at home with non-affirming family members and/or to experience disruptions in education and employment, which are common gateways to social support and mental health services for this population [20,52,62]. Further research is needed to investigate and contextualize sub-population disparities such as these, while also developing responsive care strategies for supporting LGBTQ2+ people most at risk for adverse outcomes related to alcohol/cannabis use and mental health during the pandemic and beyond.

There are strengths and limitations to this study. First, our sampling approach generated a large sample of individuals living in Canada, among whom a significant proportion (7.1%) were classified as LGBT2Q+. This larger sample is representative of the Canadian population by age, gender, region, and income, though other characteristics may not be as representative. Importantly, some groups, including racialized and Indigenous communities, are somewhat underrepresented, as we detail elsewhere [9]. In addition, the proportion of LGBT2Q+ respondents in this survey is greater than the 4% national estimate [63], though this may be due to our use of a single survey item to capture LGBTQ2+ identities, as well as our inclusion of respondents who indicated “unsure” when asked whether they identify as LGBTQ2+. This item and our survey’s partial conflation of sex/gender likely contributed to misclassification bias [64], especially for trans respondents, given current recommendations to use two-step measures for identification of trans survey respondents [65]. Self-report biases may similarly have undercounted racialized and/or Indigenous persons [66,67]. The pooled LGBT2Q+ identity measure also hindered our capacity to conduct sub-population analyses between LGBTQ2+ respondents (e.g., gay vs. lesbian vs. bisexual individuals)—this is an important limitation and an area for targeted research. Moreover, although we included Two-Spirit and non-binary trans participants in our descriptive results, the smaller sample sizes of these populations prevented us from including them in logistic regression analyses. We also excluded responses from participants who overlapped across study rounds, which may have opened the potential for bias. A key strength of our study is the repeated cross-sectional survey design, which included three rounds of data collection. However, this study design prevents us from drawing causal conclusions about the impacts of the pandemic at both individual and group levels, and in a temporal manner (i.e., as we would with longitudinal data). Sampling bias may also exist in this study, particularly if the mental health of prospective participants either facilitated or deterred their participation in the survey. Furthermore, using self-reported measures for increased substance use and mental health may have introduced self-reporting and social desirability bias. These were also single-item measures. This may be considered a study limitation; however, single-item measures of self-rated mental health do hold value in public health research, and have demonstrated associations with multi-item measures [68]. Still, future research may benefit from using multi-item, valid and reliable scales, and/or objective measures of health (e.g., stress biomarkers) to describe population-level mental health experiences more comprehensively, and to capture those that hold clinical significance.

## 5. Conclusions

This study demonstrates that, in the context of the COVID-19 pandemic in Canada, LGBTQ2+ adults are experiencing increases in alcohol and cannabis consumption, and corresponding deteriorations in self-reported mental health. Real-time and population-tailored policy responses are needed to mitigate pertinent adverse psycho-social consequences, both in the immediate and with respect to future drug-related and mental health harms, including those that may arise from dependent and/or intensive alcohol and cannabis use. Given complex and dynamic relationships between alcohol/cannabis use and mental health, as well as baseline inequities facing LGBTQ2+ adults, integrated substance use- and mental health-tailored interventions and approaches to care may hold promise for supporting this population throughout and beyond the pandemic.

## Figures and Tables

**Table 1:** Socio-demographic description of LGBTQ2+ respondents to a national Canadian repeated survey regarding mental health during the COVID-19 pandemic, 2020–2021.

	Round 1 N (%)	Round 2 N (%)	Round 3 N (%)	Total N (%)
**Age group**				
18–34 years	84 (35.3)	39 (23.6)	19 (19.2)	142 (28.3)
35–54 years	101 (42.4)	73 (44.2)	36 (36.4)	210 (41.8)
55+ years	53 (22.3)	53 (32.1)	44 (44.4)	150 (29.9)
**Gender identity ^a^**				
Cisgender man	124 (52.1)	88 (53.3)	63 (63.6)	275 (54.8)
Cisgender woman	98 (41.2)	63 (38.2)	33 (33.3)	194 (38.6)
Trans man	3 (1.3)	1 (0.6)	0 (0.0)	4 (0.8)
Trans woman	1 (0.4)	4 (2.4)	0 (0.0)	5 (1.0)
Non-binary	9 (3.8)	4 (2.4)	3 (3.0)	16 (3.2)
Two-Spirit	2 (0.8)	3 (1.8)	0 (0.0)	5 (1.0)
Not listed	0 (0.0)	2 (1.2)	0 (0.0)	2 (0.4)
**Household income ^a^, CAD ^b^**				
Under $25 k	32 (13.4)	20 (12.3)	9 (9.2)	61 (12.2)
$25 k–<$50 k	58 (24.4)	30 (18.5)	25 (25.5)	113 (22.7)
$50 k–$100 k	76 (31.9)	66 (40.7)	35 (35.7)	177 (35.5)
$100 k+	72 (30.3)	46 (28.4)	29 (29.6)	147 (29.5)
**Education completed**				
High school or less	40 (16.8)	16 (9.7)	14 (14.1)	70 (13.9)
Some college or university	39 (16.4)	41 (24.8)	15 (15.2)	95 (18.9)
College or university graduate	159 (66.8)	108 (65.5)	70 (70.7)	337 (67.1)
**Race/ethnicity ^a,b^**				
Non-racialized	149 (65.4)	117 (74.1)	68 (70.1)	334 (69.2)
Racialized (non-Indigenous)	66 (28.9)	31 (19.6)	26 (26.8)	123 (25.5)
Indigenous	13 (5.7)	10 (6.3)	3 (3.1)	26 (5.4)
**Urban/rural**				
Urban	213 (89.5)	139 (84.2)	79 (79.8)	431 (85.9)
Rural	25 (10.5)	26 (15.8)	20 (20.2)	71 (14.1)
**Pre-existing mental health condition ^a^**				
Yes	102 (43.0)	68 (41.5)	30 (30.6)	200 (40.1)
No	135 (57.0)	96 (58.5)	68 (69.4)	299 (59.9)
**Live alone**				
Yes	67 (28.2)	44 (26.7)	29 (29.3)	140 (27.9)
No	171 (71.8)	121 (73.3)	70 (70.7)	362 (72.1)
**Increased alcohol use**	56 (23.5)	46 (27.9)	21 (21.2)	123 (24.5)
**Increased cannabis use**	41 (17.2)	40 (24.2)	12 (12.1)	93 (18.5)
**Total**	238 (100.0)	165 (100.0)	99 (100.0)	502 (100)

^a^ A small number of respondents (<5%) chose not to answer some questions, which reduced the total counts for these variables. ^b^ CAD: Canadian dollar.

**Table 2:** Increased alcohol and cannabis use by mental health outcomes among LGBTQ2+ respondents to a national Canadian repeated survey during the COVID-19 pandemic, 2020–2021.

	Total Sample	Overall Coping		Change in Mental Health		Suicidal Thoughts	
	(N = 502)	Poor (N = 111)	Fairly or Very Well (N = 367)	Worse (N = 238)	Same or Better (N = 264)	Yes (N = 85)	No (N = 410)
Increased alcohol use N (%)	123 (24.5)	41 (36.9) **	79 (21.5)	79 (33.2) **	44 (16.7)	32 (37.6) **	87 (21.2)
Increased cannabis use N (%)	93 (18.5)	28 (25.2) *	61 (16.6)	59 (24.8) **	34 (12.9)	36 (42.4) **	55 (13.4)

* *p* < 0.05, ** *p* < 0.01 based on a chi-squared test. Note: A small number of respondents (<5%) chose not to answer some questions, which reduced the total counts for some variable cross-tabulations.

**Table 3:** Results of fully adjusted multivariable logistic regression models predicting poor overall coping, worse mental health, and suicidal thoughts among LGBTQ2+ respondents to a national Canadian repeated survey during the COVID-19 pandemic, 2020–2021.

	Poor Overall Coping	Worse Mental Health	Suicidal Thoughts
	Odds Ratio (95% CI)	Odds Ratio (95% CI)	Odds Ratio (95% CI)
**Survey round**			
Round 1 (reference category ^a^)	1.0	1.0	1.0
Round 2	1.66 (0.94–2.94)	1.29 (0.81–2.04)	2.72 ** (1.37–5.41)
Round 3	1.03 (0.50–2.13)	1.04 (0.61–1.79)	1.10 (0.44–2.73)
**Age**			
55+ years (reference category)	1.0	1.0	1.0
35–54 years	2.07 * (1.01–4.24)	1.11 (0.68–1.81)	3.69 ** (1.38–9.84)
18–34 years	3.07 * (1.40–6.74)	1.07 (0.59–1.92)	4.88 ** (1.68–14.18)
**Household income**			
$100 k+ (reference category)	1.0	1.0	1.0
$50 k–$100 k	1.19 (0.62–2.29)	0.65 (0.39–1.07)	0.56 (0.26–1.22)
$25 k–$50 k	0.72 (0.33–1.58)	0.65 (0.37–1.14)	0.43 (0.17–1.11)
<$25 k	3.86 ** (1.75–8.54)	1.11 (0.55–2.23)	1.16 (0.46–2.91)
**Race/ethnicity**			
Non-racialized (reference category)	1.0	1.0	1.0
Racialized (non-Indigenous)	0.90 (0.49–1.66)	0.59 * (0.37–0.96)	1.45 (0.69–3.04)
Indigenous	1.71 (0.63–4.60)	0.77 (0.31–1.90)	3.74 * (1.31–10.66)
**Gender identity**			
Man (reference category = woman) ^b^	0.81 (0.47–1.38)	0.53 ** (0.35–0.82)	1.38 (0.71–2.68)
**Pre-existing mental health condition**	1.90 * (1.10–3.29)	1.94 ** (1.24–3.03)	8.56 ** (4.06–18.05)
**Increased alcohol use**	2.28 ** (1.28–4.07)	1.98 ** (1.21–3.25)	1.19 (0.59–2.40)
**Increased cannabis use**	0.98 (0.52–1.83)	1.45 (0.84–2.51)	2.30 * (1.16–4.55)
**Nagelkerke R Square**	0.216	0.164	0.365

^a^ The reference category is the group in relation to which remaining categories for that variable are compared. ^b^ Non-binary and Two-Spirit respondents were excluded from the regression analyses due to low numbers; binary trans participants were included in their self-identified gender category. * *p* < 0.05; ** *p* < 0.01.

## Data Availability

The data analyzed during the current study are not publicly available due to them containing information that could compromise research participant privacy and consent, but they are available from the corresponding author on reasonable request.
